# Prospective Randomized, Double-Blind, Placebo-Controlled Study of a Standardized Oral Pomegranate Extract on the Gut Microbiome and Short-Chain Fatty Acids

**DOI:** 10.3390/foods13010015

**Published:** 2023-12-19

**Authors:** Raja K. Sivamani, Mincy Chakkalakal, Adrianne Pan, Dawnica Nadora, Mildred Min, Ashley Dumont, Waqas A. Burney, Cindy J. Chambers

**Affiliations:** 1 Integrative Skin Science and Research, Sacramento, CA 95815, USA; 2 Department of Dermatology, University of California-Davis, Sacramento, CA 95616, USA; 3 College of Medicine, California Northstate University, Elk Grove, CA 95757, USA; 4 Pacific Skin Institute, Sacramento, CA 95815, USA; 5 Coastal Thyme Holistic Skin and Wellness, Plymouth, NH 03801, USA; ashley.dumont16@gmail.com

**Keywords:** pomegranate, Pomella, ellagitannins, punicalagin, gut microbiome, gut health, short-chain fatty acids, urolithins

## Abstract

*Punica granatum* L., commonly known as the pomegranate, is an abundant source of polyphenols, including hydrolyzable ellagitannins, ellagic acid, anthocyanins, and other bioactive phytochemicals shown to be effective in defending against oxidative stress, and has immunomodulatory activities. Ellagitannins, and their hydrolyzed product ellagic acid, interact with the gut microbiota to yield secondary metabolites known as urolithins that may have health benefits. The objective of this study was to determine the effects of supplementation with a standardized punicalagin-enriched pomegranate extract, Pomella^®^ (250 mg), on the gut microbiome, circulating short-chain fatty acids, and gut microbial-derived ellagitannin metabolite urolithins. A randomized, double-blind, placebo-controlled study was conducted over 4 weeks on healthy volunteers aged 25–55 years. Subjects were randomly assigned to receive either an oral supplement containing 75 mg of punicalagin or an oral placebo. Stool sample collection and venipuncture were performed to analyze the gut microbiome, SCFAs, and urolithin. There was no significant change in the gut microbial diversity in both cohorts after 4 weeks of intervention, but there was a significantly increased relative abundance of *Coprococcus eutectus*, *Roseburia faecis*, *Roseburia inullnivorans*, *Ruminococcus bicirculans*, *Ruminococcus calidus*, and *Faecalibacterium prausnitzii*. Pomegranate extract (PE) supplementation led to the augmentation of circulating propionate levels (*p* = 0.02) and an increasing trend for acetate levels (*p* = 0.12). The pomegranate extract (PE) supplementation group had an increased level of circulating urolithins compared to the placebo group (6.6% vs. 1.1%, *p* = 0.13). PE supplementation correlated with shifts in the gut microbiome and with higher circulating levels of propionate and acetate. Further studies should explore the implications in larger cohorts and over a longer duration.

## 1. Introduction

The role of diet and nutrition in managing health has become a key area of interest for both practitioners and the general public. For centuries, *Punica granatum* L., or the pomegranate fruit, has been widely used as a remedy to treat various ailments in folk medicine. Furthermore, pomegranates have been labeled as a “superfood” due to their antioxidant properties and they are a rich source of vitamins, minerals, polyphenols, sugars, organic acids, and fatty acids, along with other phytonutrients. As a result, a growing number of research studies have examined the potential health benefits of pomegranate polyphenols as a functional food [1,2,3,4,5]. 

Although many preclinical studies and clinical studies have evaluated the health benefits of different components of pomegranate juices and extracts, studies examining the effects of pomegranate extracts on the gut microbiota are in the early stages. For example, pomegranate extract (450 mg) and pomegranate juice (25 µg/mL) increased the relative abundance of beneficial bacteria such as *Bifidobacterium* by 20% and 15%, respectively, in an in vitro fecal culture study. Both agents also increased the mean counts of *Lactobacillus* [3,5]. Another study reported an increase in *Bifidobacterium* after 4 weeks of pomegranate peel extract (6 mg/day) supplementation in high-fat diet-induced obese mice [4]. The prebiotic effect on *Bifidobacterium* and *Lactobacillus* may offer increased protection against the overgrowth of external and internal microorganisms which are implicated in disease, aging, and cancer [1]. Moreover, the enrichment of these species may be modulated by urolithin (UA) metabolites, which are derived from the hydrolysis of ellagitannins and ellagic acid by the gut microbiota. These metabolites are considered beneficial as they are preclinically implicated in proper mitochondrial function and may be beneficial against gut, neurodegenerative, or metabolic disorders [2,3,5]. Furthermore, we previously reported that oral pomegranate extract can improve the appearance of skin and shift the skin’s biophysical measures [6]. However, clinical studies examining the influence of the phytoconstituents of pomegranate on the gut microbiota and its secondary metabolites, such as urolithins and short-chain fatty acids, have not been explored. 

In this study, we evaluated the effects of a standardized punicalagin-enriched pomegranate extract (250 mg) on the gut microbiome and circulating short-chain fatty acids after four weeks of oral supplementation in healthy volunteers.

## 2. Material and Methods

### 2.1. Study Design and Recruitment 

**Figure 1:**
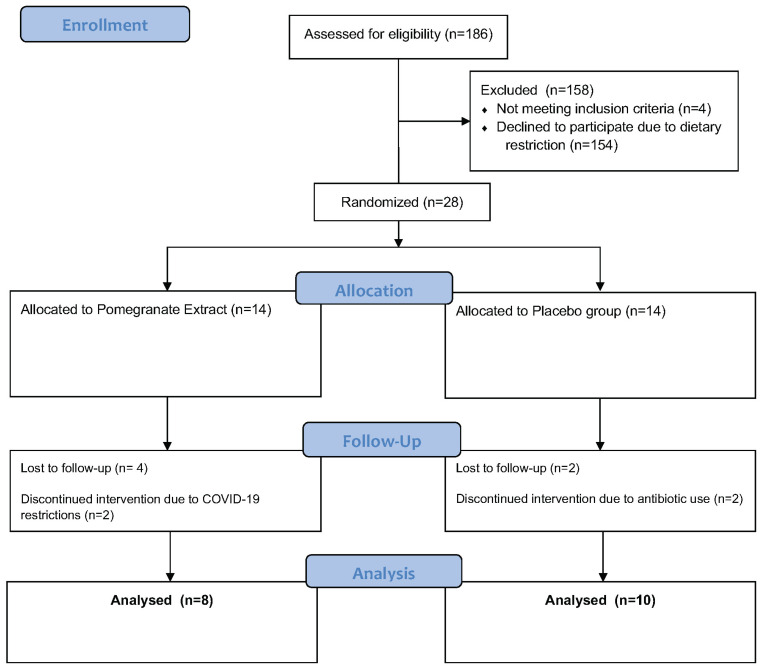
CONSORT diagram schematic for the study.

This four-week, double-blind, placebo-controlled study was conducted between November 2020 and March 2021 as a pilot clinical trial [6]. This study was approved by the Institutional Review Board (IntegReview Ltd., Austin, TX, USA) and registered at www.clinicaltrials.gov (NCT04596722). Written informed consent was obtained from all participants prior to any participation. Healthy males and females living in the Sacramento region, ages 25–55, were recruited and screened for eligibility. All study procedures were performed at Integrative Skin Science and Research in Sacramento, CA. A total of 186 individuals were assessed for eligibility, of which 28 subjects met inclusion criteria and were randomized and pre-allocated into the following two study groups using a computer-based randomization generator with blinded sealed envelopes. The pomegranate extract (PE) group (n = 14) received an oral supplement (Pomella^®^, Verdure Sciences, Inc. Noblesville, IN, USA) and the control group (n = 14) received an oral placebo for daily consumption for four weeks [6]. The Pomella^®^ supplement utilized in this study was provided by Verdure Sciences, Inc. (Noblesville, IN, USA) and consisted of the following ingredients: pomegranate whole fruit extract containing 75 mg punicalagin. The placebo supplement utilized in this study contained the following ingredients: 97.67% maltodextrin, 0.10% tartazine color, 0.22% brown color, and 2.01% aerosol. In the control group, 2 subjects were lost to follow-up after screening and 2 discontinued the study intervention due to antibiotic use. In the PE group, 4 subjects were lost to follow-up after screening and 2 discontinued the study intervention due to concerns related to COVID-19 [6]. The flow of participants throughout the study is depicted in Figure 1.

### 2.2. Inclusion and Exclusion Criteria

Individuals with a known allergy to the study agents were excluded from the study. Participants were asked to discontinue the use of other nutritional supplements, including prebiotics and probiotics, at least one month prior to enrollment and for the duration of the study. Individuals who had used systemic, injected, or oral antibiotics within 6 months or those who had started a new diet such as the ketogenic diet within one month were excluded. Current smokers and individuals who had smoked within the past year or who had a five-year pack history were excluded. Individuals with a history of the following conditions were also excluded: malignancy; cancer (excluding non-metastatic cancer); gastrointestinal inflammatory diseases; epilepsy; immunologic or infectious diseases such as hepatitis, tuberculosis, human immunodeficiency virus, acquired immunodeficiency syndrome, lupus, or rheumatoid arthritis [6]. Other exclusion criteria included the current use of medications that alter blood lipids, such as statins and anti-hyperlipidemic medications; individuals with a body mass index higher than 35 kg/m^2^, individuals who adhered to the vegan diet; individuals who refused to shave facial hair which may interfere with image collection and assessment; individuals who were participating in or had participated in an intervention-based facial study two weeks prior to the baseline visit; women who had been pregnant in the last three months, were currently pregnant, preparing to be pregnant, or lactating; and prisoners and adults who were unable to provide consent on their own [6]. 

### 2.3. Dietary Restrictions and Microbiome Sampling

Participants were asked to adhere to the following set of dietary restrictions to reduce the confounding effects of polyphenolic-rich foods. Participants were asked to reduce coffee intake to, at most, an eight-ounce cup of coffee and to only consume a cup of berries and a cup of fermented dairy products per week throughout the duration of the study. This diet also included the complete restriction of chocolate, alcohol, tea, and pomegranate or pomegranate-containing drinks for the four weeks of the study [6]. 

Stool samples were collected from subjects at baseline and 4 weeks using a manually assembled kit for gut microbiome sampling. All samples were stored at −80 °C and shipped according to the instructions provided by CosmosID (Germantown, MD, USA).

### 2.4. Stool DNA Extraction and Whole Genome Sequencing 

Taxonomic and functional analyses of the WGS stool samples were performed by CosmosID (Germantown, MD, USA). DNA from fecal samples was isolated using a QIAGEN DNeasy PowerSoil Pro Kit (QIAGEN Sciences, Germantown, MD, USA), according to the manufacturer’s protocol. Extracted DNA samples were quantified using Qubit 4 fluorometer and Qubit™ dsDNA HS Assay Kit (Thermofisher Scientific, Waltham, MA, USA).

#### 2.4.1. Library Preparation

DNA libraries were prepared using a Nextera XT DNA Library Preparation Kit (Illumina, San Diego, CA, USA) and IDT Unique Dual Indexes with total DNA input of 1ng. Genomic DNA was fragmented using a proportional amount of Illumina Nextera XT fragmentation enzyme. Unique dual indexes were added to each sample followed by 12 cycles of PCR to construct libraries. DNA libraries were purified using AMpure magnetic Beads (Beckman Coulter, Brea, CA, USA) and eluted in QIAGEN EB buffer. DNA libraries were quantified using Qubit 4 fluorometer and Qubit™ dsDNA HS Assay Kit. Libraries were then sequenced on an Illumina HiSeq 4000 platform 2 × 150 bp [6].

#### 2.4.2. Bioinformatics Analysis of Stool Samples

Unassembled sequencing reads to a depth of approximately 6M were directly analyzed by CosmosID-HUB Micro-biome Platform (CosmosID Inc., Germantown, MD, USA) as described elsewhere for multi-kingdom microbiome analysis, the profiling of antibiotic resistance and virulence genes, and quantification of organisms’ relative abundance [7,8,9,10]. Briefly, the system utilizes curated genome databases and a high-performance data-mining algorithm that rapidly disambiguates hundreds of millions of metagenomic sequence reads into the discrete microorganisms engendering the particular sequences. Similarly, the community resistome and virulome and the collection of antibiotic resistance and virulence-associated genes in the microbiome were also identified by querying the unassembled sequence reads against the CosmosID curated antibiotic resistance and virulence-associated gene databases [6].

### 2.5. Short-Chain Fatty Acid Measurements

Blood was collected from participants by venipuncture, the collection tubes were centrifuged, and the supernatant plasma was selectively collected and stored at −80 °C until they were shipped for analysis. Short-chain fatty acids were measured by Creative Proteomics (Shirley, NY, USA). Samples were diluted in water containing labeled internal standards. The free short-chain fatty acids were derivatized using methyl chloroformate in 1-propanol, yielding propyl esters, before subsequent liquid–liquid extraction into hexane and analysis on an SLB-5 ms (30 × 0.25 mm × 1.0 µm) column from Supelco, Sigma-Aldrich, St. Lousi, MO, USA. Agilent 6890 GC coupled to an Agilent 5973 MSD detector (Agilent Technologies, Inc., Santa Clara, CA, USA) in SIM mode was used for short-chain fatty acid analysis. The analytes were quantified using 8-point calibration curves. The chromatograms for the short-chain fatty acids can be visualized in Figure S1. 

### 2.6. Urolithin a Measurements

After sample preparation and LC-MS analysis, a standard substance of urolithin A (Sigma-Aldrich, St. Louis, MO, USA) was dissolved in an internal standard (IS) solution of dopamine-D4 in acetonitrile. This solution was serially diluted to have 10-point calibration solutions in a concentration range of 0.005 to 16 ng/mL. Each plasma sample was thawed on ice. After vortex-mixing, 100 μL of each sample was mixed with 100 μL of the IS solution and 200 μL of acetonitrile. The mixture was vortex-mixed for 30 s at 3000 rpm and then sonicated in an ice-water bath for 5 min. The samples were clarified by centrifugation at 15,000× *g* and 5 °C for 5 min. A total of 320 μL of the clear supernatant of each sample was taken out and dried under a nitrogen gas flow at 30 °C. Then, 20 μL of acetonitrile was added to the residue of each sample. Subsequently, each of the samples or 20 μL of each calibration solution was mixed in turn with 80 μL of a dansyl chloride solution and 60 μL of borate buffer. The mixtures were allowed to react at 40 °C for 30 min. UPLC-MRM/MS: After reaction, 10 μL of each resultant solution was injected to run UPLC-MRM/MS on a Waters Acquity UPLC system coupled to a Sciex QTRAP 6500+ mass spectrometer (AB Sciex LLC, Framingham, MA, USA) operated in the positive-ion mode. LC separation was carried out on a C18 LC column (2.1 × 100 mm, 1.7 μm) from Waters Corporation, Milford, MA, USA, with 0.1% formic acid in water and 0.1% formic acid in acetonitrile as the binary solvents for gradient elution (50% to 85% B in 15 min) at 50 °C and 0.30 mL/min. 

Concentrations of urolithin A detected in the samples were calculated with internal standard calibration by interpolating the linear-regression calibration curve of urolithin A, constructed with the data acquired from injections of the calibration solutions, with the analyte to internal standard peak area ratios measured from injections of the sample solutions.

### 2.7. Statistical Analysis

Parametric data results are presented as the mean ± standard deviation. Non-parametric data results are presented as the median ± 95% confidence interval. The statistical analysis for parametric data was performed by using Student’s t-test to assess the within-group (two-tailed, paired) and between-group (two-tailed, unpaired) differences [6]. A chi-square test with Yates’ correction was performed to analyze non-parametric data. Yates’s correction was applied to reduce the overestimation of statistical significance for the survey results, which were composed of responses that had an expected count smaller than five [6]. Statistical significance was set at *p* < 0.05. *p* < 0.15 was considered a trend. Differential microbiome abundance was determined using Linear Discriminant Analysis effect size (LEfSe) analysis at all taxonomic levels. Differential feature and organism abundance between groups were calculated by using Kruskal–Wallis sum-rank test, Wilcoxon rank-sum test, and Linear Discriminant Analysis (LDA). All features shown meet *p* ≤ 0.05 for Kruskal–Wallis and Wilcoxon tests and have an LDA score where the effect size ≥ 2.0. The statistical significance was determined by using the Wilcoxon rank-sum test. *p* < 0.05 was considered statistically significant and *p* values up to 0.15 or less were considered trends [6]. 

## 3. Results

### 3.1. Subject Demographics

A total of 28 subjects were enrolled and 15 females and 3 males completed the study. In the control group, there were 10 participants (F = 9, M = 1); their average age was 42.3 ± 10.5 and the average BMI was 24.4 ± 4.5. In the PE group, there were eight participants (F = 6, M = 2); their average age was 40 ± 11.2 and the average BMI was 24.6 ± 4.6 [6]. 

### 3.2. Gut Microbiome 

**Figure 2:**
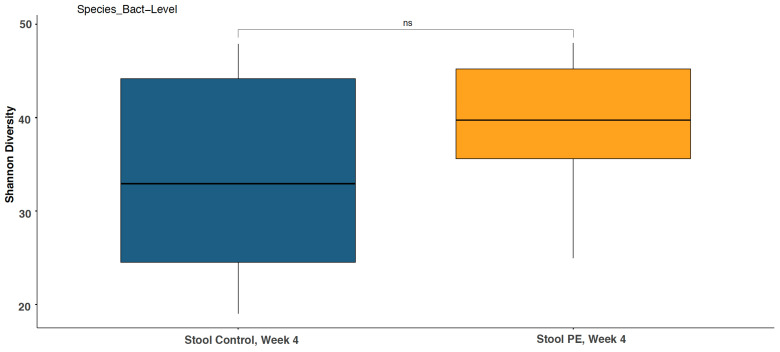
Stool Shannon diversity at the bacterial species level was not significantly changed with PE supplementation compared to the control group at week 4. ns = not statistically significant.

No significant differences in alpha diversity (the Shannon diversity index) were observed at the genus, phylum, species, and strain levels at baseline between the PE group and the control group (*p* > 0.05). Supplementation with the PE for 4 weeks did not shift the Shannon diversity index compared to the placebo from baseline at the genus, phylum, species, and strain levels (Figure 2). 

**Figure 3:**
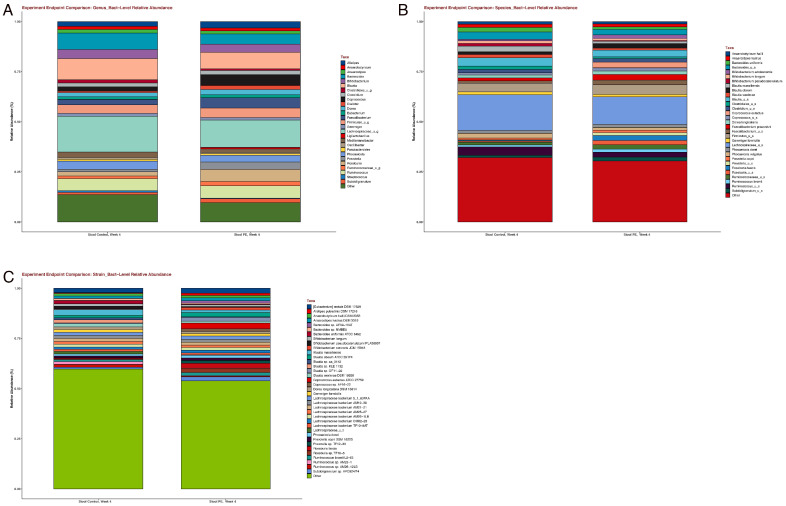
Shifts in the gut microbiome were noted in the (**A**) genus, (**B**) species, and (**C**) strain levels after four weeks of PE supplementation.

**Figure 4:**
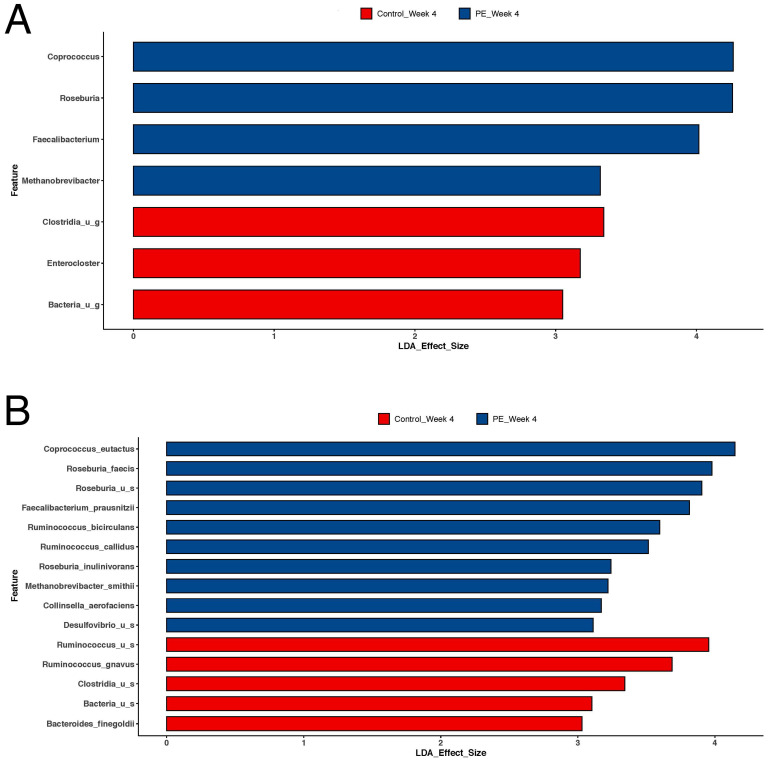
Lefse analysis of the microbiome comparing shifts in the microbiome after 4 weeks of PE supplementation compared to placebo (control) supplementation. Results are shown for both (**A**) genus and (**B**) species. LDA effect size > 2 was considered statistically significant. There were significant increases in *Coprococcus eutectus*, *Roseburia faecis*, *Roseburia inullnivorans*, *Ruminococcus bicirculans*, *Ruminococcus calidus*, *Faecalibacterium prausnitzii*, *Methanobrevibacgter smithii*, and *Collinsella aerofaciens*.

However, supplementation in the PE group was associated with shifts in individual bacteria at the genus, species, and strain levels (Figure 3). PE supplementation led to an increased relative abundance in the following bacteria (Figure 4): *Coprococcus eutectus*, *Roseburia faecis*, *Roseburia inullnivorans*, *Ruminococcus bicirculans*, *Ruminococcus calidus*, *Faecalibacterium prausnitzii*, *Methanobrevibacgter smithii*, and *Collinsella aerofaciens*.

### 3.3. Gut Microbiome Functional Analysis 

**Figure 5:**
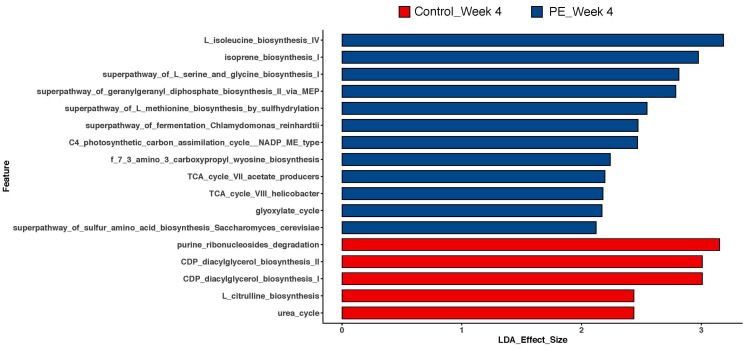
LEfSe analyses of MetaCyc metabolic pathways after 4 weeks of PE or placebo supplementation from the gut microbiome. LDA effect size > 2 was considered statistically significant.

Functional analyses of the genes present in the gut after 4 weeks of supplementation with either PE or placebo are shown in Figure 5. PE supplementation augmented the presence of genes for the synthesis of several amino acids, including L-isoleucine, L-serine, L-glycine, and L-methionine. The presence of genes for the synthesis of geranylgeranyl diphosphate was augmented in the PE-supplemented group. The PE-supplemented group was found to have more genes that support a catabolic state for the TCA cycle along with genes for the activity of the glyoxylate cycle. Finally, there was the augmentation of the gene for the super pathway of sulfur amino acid biosynthesis which is specific to the activity of *Saccharomyces cerevisiae*.

### 3.4. Plasma Short-Chain Fatty Acids

**Figure 6:**
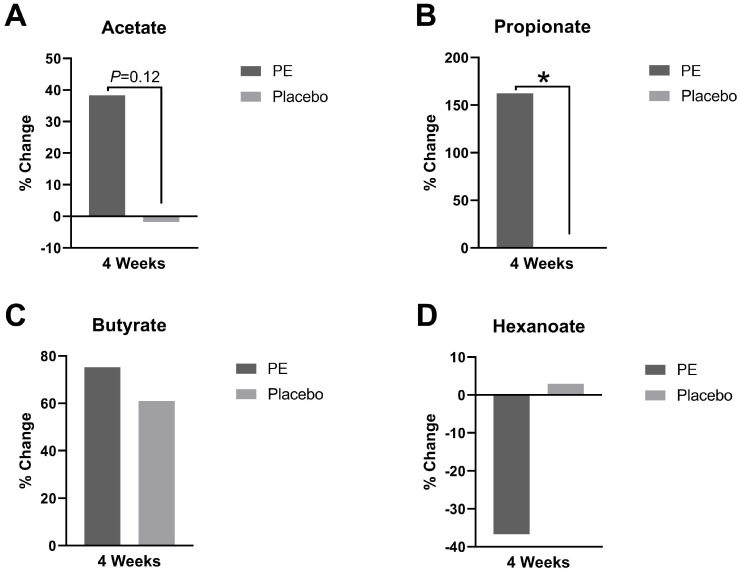
Plasma short-chain fatty acid levels were quantified and presented as percent change from baseline after 4 weeks of supplementation with PE or placebo. The plasma levels of (**A**) acetate, (**B**) propionate, (**C**) butyrate, and (**D**) hexanoate were analyzed. * *p* < 0.05.

The plasma levels of short-chain fatty acids before and after PE or placebo supplementation were measured, and there were notable differences in the short-chain fatty acids (Figure 6). There was a 162% increase in the propionate level (*p* = 0.02) and a 38% increase in the acetate level (*p* = 0.12) after PE supplementation. There were no significant changes or trends in butyrate or hexanoate levels after PE supplementation compared to those in the placebo. 

### 3.5. Plasma Urolithin A

**Figure 7:**
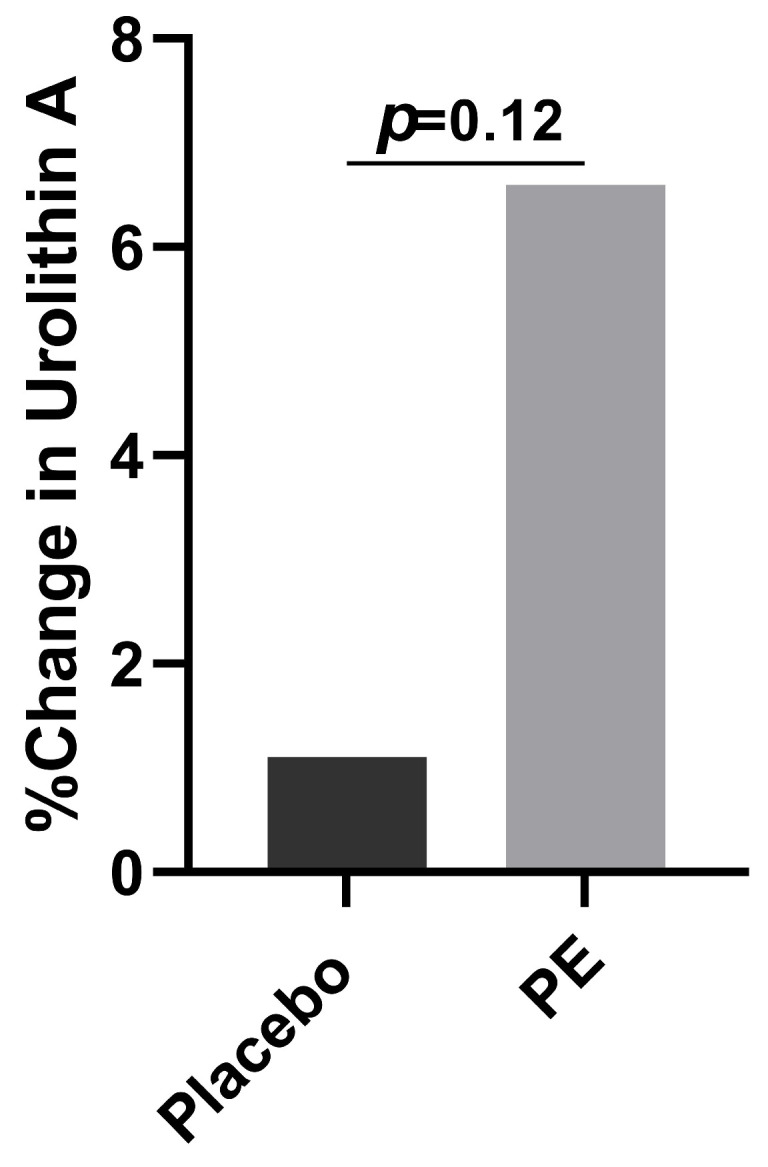
Percent change from baseline of plasma urolithin A concentrations in both the PE and the placebo groups after 4 weeks of supplementation.

Those in the PE group had a greater increase in their urolithin A levels compared to placebo supplementation (6.6% vs. 1.1%, *p* = 0.13, Figure 7). When considering how many of the participants had at least a 10% increase in their urolithin A plasma levels, the PE supplementation group had a greater likelihood of achieving a 10% increase compared to those in the placebo group (*p* = 0.06). 

## 4. Discussion

Our study revealed that the oral supplementation of Pomella^®^, a punicalagin-standardized PE extract (75 mg of punicalagins), leads to significant shifts in the gut microbiota, increases the circulating plasma levels of short-chain fatty acids such as propionate and acetate, and increases the plasma levels of urolithin A levels. 

### 4.1. Gut Microbiome Shifts 

The composition of the gut microbiome may be influenced by diet, disease, and drugs. PE supplementation shifted the gut microbiome after 4 weeks and there were several patterns that emerged. There was no shift in the overall diversity of the gut. However, there were shifts in the individual genera and species. For example, there was an increase in species such as *Coprococcus eutectus*, *Roseburia faecis*, *Roseburia inullnivorans*, *Ruminococcus bicirculans*, *Ruminococcus calidus*, *Faecalibacterium prausnitzii*, *Methanobrevibacter smithii*, and *Collinsella aerofaciens* in the PE cohort, which indicates that pomegranate ellagitannins induce shifts in the bacteria that may influence overall health through the modulation of short-chain fatty acids, secondary metabolites, and urolithin A synthesis. 

There was an increased relative abundance in short-chain fatty acid (SCFA) producers and this correlates with the increase in the circulating plasma levels of the SCFAs, such as acetate and propionate, that we measured. For example, *Roseburia* and *Faecalibacterium prausnitizii* are producers of short-chain fatty acids such as butyrate and acetate and may promote the growth of other SCFA-producing microbes [11,12]. *Coprococcus eutectus* has been shown to produce SCFAs such as butyrate and propionate [11]. SCFAs serve an anti-inflammatory function locally in the gut and as mediators to reduce inflammation distant to the gut [13]. Moreover, they reduce the overactivation of the immune system [14]. 

The increase in the relative abundance of the genus *Ruminococcus* may further explain previously noted cardiovascular benefits associated with pomegranate extract consumption [15,16,17]. For example, the presence and relative abundance of *Ruminococcus* has been associated with decreased cardiovascular risk in an obese population [18]. 

### 4.2. Functional Predictive Shifts in the Gut Microbiome 

Apart from the shifts in the gut microbiota, the functional predictive analysis of the microbial genes present in the gut revealed interesting shifts. The abundance of synthetic genes that became more abundant after PE supplementation suggests that the gut microbiota were activated in response to the intake of PE. For example, while there was not an increased overall relative abundance in the presence of *Saccharomyces cerevisiae*, the genes present for its increased activity in the synthesis of methionine and cysteine [19] were augmented after PE supplementation. Our findings suggest that PE supplementation is not limited to shifts in the microbiome, but they shift the activity of certain microbes. Furthermore, the shifts are not limited to bacteria and also involved fungi, as evidenced by the findings with *Saccharomyces cerevisiae*. 

The functional predictive analyses also revealed an augmentation of microbes with the TCA cycle VII (acetate producers) and glyoxylate cycle genes (Figure 5). The glyoxylate cycle is activated in bacteria that are grown in the presence of acetate [20] and correlates with the increasing trend in acetate production after PE supplementation (Figure 6A). Our results show that the functional analyses of the gut correlate with circulating SCFAs and further support the notion that PE supplementation augments circulating SCFAs through modulation of the gut microbiome as one of the mechanisms. 

The expression of the genes for the L-isoleucine synthesis pathway suggests that the gut bacteria were stimulated to a state that suggests readiness to synthesize isoleucine. This branched-chain amino acid is an essential amino acid that contributes to muscle growth and enhanced glucose control when taken in moderation [21]. Our findings that the presence of the genes for the synthesis of isoleucine is upregulated in the pomegranate extract group suggest another mechanism by which the extract may support the skin. Isoleucine and the branched amino acid augment collagen synthesis when supplemented in mice [22]. Future studies should explore if pomegranate extract supplementation may augment circulating isoleucine levels. Additionally, these studies should explore how PE supplementation may augment collagen and skeletal muscle stimulation distinctly from its antioxidant effects. 

### 4.3. Implications of Gut-Microbiome-Related Secondary Metabolites or Co-Factor Synthesis

The increase in *Coprococcus eutectus* may have other benefits outside of their SCFA production. A study in mice showed that the presence of *Coprococcus eutectus* was associated with increased motivation and ability to exercise [23]. Metabolites produced by *C. eutectus* act as endocannabinoids that can stimulate the CB1 and TRPV1 receptors to produce dopamine during exercise [23]. Pomegranate extract intake has previously been shown to increase exercise ability in humans [24], and one of the mechanisms of action may be through shifting the microbiome and its metabolome. 

PE supplementation increased the presence of *Roseburia*, which is involved in the biosynthesis of multiple vitamin-related co-factors. For example, the *Roseburia* genus is involved in the synthesis of multiple B vitamins and *Roseburia inulinivorans* is involved in folate synthesis [12]. 

### 4.4. Short-Chain Fatty Acid Augmentation

The increases in the circulating levels of propionate (162%) and acetate (38%) mechanistically show that the impact of PE supplementation goes beyond the gut. This has important implications for the modulation of conditions that may be sensitive to shifts in SCFAs. For example, deficiencies of acetate have been measured in acne [25] and deficiencies in propionate have been reported in obesity and diabetes [26]. 

Accordingly, the augmentation of short-chain fatty acids has been shown to improve inflammatory and metabolic health. An increase in short-chain fatty acids has been associated with the treatment of hidradenitis suppurativa [27]. Probiotic-induced increases in acetate have been shown to improve acne [25]. SCFA augmentation has also been associated with cardiometabolic health [28]. The observed increases in propionate and acetate suggest that future studies on PE extract should evaluate health conditions that are characterized by SCFA deficiencies. These conditions may span inflammatory and metabolic diseases that involve the skin and overall well-being. With a growing need to find alternatives to the chronic use of antibiotics, a clinical study of the impact of standardized PE extracts on acne and hidradenitis suppurativa would be warranted. 

We previously reported the skin-related benefits of PE extract consumption [6], and this study expands our previous findings to the gut- and health-related metabolic benefits. One of the advantages of utilizing nutrition and dietary supplement-based approaches to skin care is that there are benefits that go beyond the skin, including the potential to modulate the gut microbiome. Future studies that evaluate the role of PE supplementation in modulating SCFAs would further clarify the role of PE extract consumption on the gut microbiome and other metabolites.

Future studies should focus on whole genome analysis of the genes that are present in the identified microorganisms and should correlate them against pathway analysis approaches to better correlate SCFAs with shifts in the gut microbiome. 

### 4.5. Urolithin A 

Our study found that PE supplementation led to a greater increase in plasma urolithin A concentrations than the placebo intervention. Urolithins are bioactive compounds produced by gut bacteria as a result of ellagitannin and ellagic acid-rich food metabolism, such as in pomegranate extract consumption [29]. Urolithins as a class have been shown to have anti-cancer [30], neuroprotective [31], cardioprotective [32], and anti-inflammatory [33] properties. Some of the bacteria involved in urolithin production include the *Lactobacillus*, *Roseburia*, *Fusobacterium*, *Streptococcus*, *Slackia*, and *Bacillus* genera [34]. Thus, the increased abundances of *Roseburia* found in our study may correlate with the increased plasma urolithin A levels observed after PE supplementation. While literature on other types of urolithins is rapidly emerging, urolithin A has been shown to notably protect against inflammatory intestinal barrier dysfunction. It is likely that PE supplementation led to an increase in the anti-inflammatory urolithin A by two mechanisms. First, the increased consumption of the ellagitannin punicalagin likely led to higher metabolite production due to the involvement of ellagitannins in the synthesis of urolithins. Second, it is possible that PE supplementation led to an increase in microorganisms involved in urolithin production. Future studies should look at how PE supplementation alters the plasma levels of the various types of urolithins, such as urolithin M5, B, C, and D, and should correlate these changes to the gut microbiome and overall health. 

### 4.6. Limitations

Our study had several limitations. This study was limited to a four-week interventional period, and so we are not able to comment on the long-term effect of supplementation. The study imposed many limitations on diet and dietary intake, but this reduces the number of confounders. This study was a pilot study with a restricted number of participants and with a predominance for the recruitment of women. Thus, it remains unclear whether the effects of PE supplementation are more or less responsive based on gender. The results here warrant future studies with an expanded cohort of participants that should include more men as part of the study group. Since ellagitannins are water-soluble polyphenols that are easily absorbed in the digestive tract, the resulting effects of PE supplementation may be short-lived. More research is needed to determine the optimal frequency of dosing for PE supplements. 

## 5. Conclusions

In this study, we assessed changes in the gut microbiome and circulating short-chain fatty acids after a four-week oral supplementation of punicalagin-enriched pomegranate extract in healthy subjects. Our results demonstrate a significant increase in the abundance of multiple short-chain producing bacteria in the gut microbiome of the PE group along with an increase in the circulating acetate and propionate levels. Our findings suggest that punicalagin standardized pomegranate extract consumption supports a healthier gut and gut–body communication. Future studies should explore the utility of PE ingestion for clinical conditions such as acne and hidradenitis suppurativa.

## Data Availability

Data is contained within the article and Supplementary Materials.

## Supplementary Materials

The following supporting information can be downloaded at: https://www.mdpi.com/article/10.3390/foods13010015/s1, Figure S1: Chromatograms for Short-Chain Fatty Acids: Acetic Acid, Hexanoic Acid, Butyric Acid, Propionic Acid, Valeric Acid.
